# Adverse pregnancy outcomes during the COVID-19 lockdown. A descriptive study

**DOI:** 10.1186/s12884-021-04221-6

**Published:** 2021-11-10

**Authors:** Eman F. Badran, Rula M. Darwish, Yousef Khader, Rama AlMasri, Mira Al Jaberi, Mohammad AlMasri, Farah AlSa’di, Leen abu Yosef, Noor al-Badaineh

**Affiliations:** 1 Department of Pediatrics, School of Medicine, The University of Jordan, PO Box 11492, Amman, Jordan; 2 Department of Pharmaceutics and Pharmaceutical Technology, School of Pharmacy, The University of Jordan, Amman, Jordan; 3 Department of Public Health, Jordan University of Science and Technology, Irbid, Jordan

**Keywords:** COVID-19, Extremely low birth weight, Lockdown, Neonates

## Abstract

**Background:**

The ongoing spread coronavirus disease worldwide has caused major disruptions and led to lockdowns. Everyday lifestyle changes and antenatal care inaccessibility during the coronavirus disease 2019 (COVID-19) pandemic have variable results that affect pregnancy outcomes. This study aimed to assess the alterations in stillbirth, neonatal-perinatal mortality, preterm birth, and birth weight during the COVID-19 national lockdown.

**Methods:**

We used the data from the Jordan stillbirths and neonatal death surveillance system to compare pregnancy outcomes (gestational age, birth weight, small for gestational age, stillbirth, neonatal death, and perinatal death) between two studied periods (11 months before the pandemic (May 2019 to March 2020) vs. 9 months during the pandemic (April 2020 to March 1st 2020). Separate multinomial logistic and binary logistic regression models were used to compare the studied outcomes between the two studied periods after adjusting for the effects of mother’s age, income, education, occupation, nationality, health sector, and multiplicity.

**Results:**

There were 31106 registered babies during the study period; among them, 15311 (49.2%) and 15795 (50.8%) births occurred before and during the COVID-19 lockdown, respectively. We found no significant differences in preterm birth and stillbirth rates, neonatal mortality, or perinatal mortality before and during the COVID-19 lockdown. Our findings report a significantly lower incidence of extreme low birth weight (ELBW) infants (<1kg) during the COVID-19 lockdown period than that before the lockdown (adjusted OR 0.39, 95% CI 0.3-0.5: *P* value <0.001)

**Conclusions:**

During the COVID-19 lockdown period, the number of infants born with extreme low birth weight (ELBW) decreased significantly. More research is needed to determine the impact of cumulative socio-environmental and maternal behavioral changes that occurred during the pandemic on the factors that contribute to ELBW infants.

## Background

Numerous coronavirus outbreaks posing a great public health threat have occurred throughout the years, including the Middle East respiratory syndrome and severe acute respiratory syndrome (SARS-CoV). The current alarming worldwide spread of SARS-CoV-2 has caused worldwide disarray, with the World Health Organization (WHO) declaring it a public emergency in January 2020 [1].

The coronavirus disease (COVID-19) pandemic has caused an international outcry, leading to lockdowns, as well as health care and economic crises, in many countries [2]. Globally, numerous countrywide methods have been implemented to limit the virus spread, including social distancing and quarantine, as well as partial or complete lockdowns.

Individual studies and systematic reviews are increasingly giving evidence on the influence of other recent viral epidemics on pregnancy outcomes. According to the current literature, pregnancy may worsen the course of COVID-19 infection when compared to non-pregnant women of the same age. COVID-19 has been linked to an increase in obstetric complications like fetal distress, cesarean delivery, and both iatrogenic and spontaneous preterm birth [3]. The reported mechanism is vertical transmission of SARS-CoV-2, either in utero via the hematogenous transplacental route, resulting in an increased rate of decidual arteriopathy and other features of maternal vascular malperfusion. It may be rarely transmitted as intrapartum ascending route with aspiration of amniotic fluid or even the early postnatal period [3–5].

In the context of the 2014-2019 Ebola outbreak in West Africa, evidence suggests that nearly all pregnant women who contract Ebola have a negative pregnancy outcome. Perinatal mortality among Ebola-infected women's infants is extremely high, with only a small percentage surviving the neonatal period [6]. A comprehensive review and meta-analysis published in 2016 found a positive relationship between symptomatic dengue virus infections during pregnancy, preterm delivery, and low birth weight. Moreover, an increase in the risk of stillbirth was recorded in Brazil (2006–2012) [6–8]. A causal relationship between prenatal Zika Virus (ZIKV) infection and a variety of congenital brain abnormalities, including microcephaly. In this context, other negative fetal outcomes (such as preterm, low birth weight, small-for-gestational-age, and fetal death) related with ZIKV infection during pregnancy have not been well quantified [9]. There is limited data on the impact and likelihood of MERS-CoV during pregnancy emerged in 2012. There was a higher rate of fetal death (27%) among 12 reported pregnant women with MERS women compared to 0% among COVID-19 [10].

With an increasing number of pregnant women being diagnosed with COVID-19 worldwide, our understanding of the effect of COVID-19 on fetal outcomes at population level remains limited. As healthcare services become overwhelmed by COVID-19 cases and means of addressing them, there is a threat of the already fragile infrastructure collapsing. There are concerns regarding whether these services can provide adequate antenatal care, especially in low- and middle-income countries [3].

During the early months of 2020, COVID-19 caused a worldwide healthcare emergency. Among other countries, it affected Jordan, a Middle Eastern upper-middle-income country with approximately 10 million inhabitants in 2020 [11]. The state of emergency and lockdown measures implemented by the government due to the pandemic caused limitations and challenges for healthcare providers. The restricted mobility of patients, including pregnant women and medical staff, as well as the lack of accessibility to diagnostic tools, were among the obstacles faced. These measures warranted further expansion of telemedicine use to guarantee that patients, including pregnant women, retain access to health care providers and attend regular follow-up appointments. Moreover, round-the-clock access to emergency departments was guaranteed [12–15].

Given the novelty of the virus, only modeling studies have investigated whether the COVID-19 pandemic affects mothers and their babies, with many of them anticipating decreased accessibility to health services. Several studies have attributed maternal mortality during the COVID-19 pandemic to comorbidities; on the other hand, neonatal mortality has been attributed to prematurity rather than infection [16–18]. The pandemic could affect the neonatal mortality rate due to the anticipated decline in health care services and possibly due to fear of mothers visiting health care facilities due to COVID-19 [19].

Prematurity is the leading cause of death in children aged < 5 years [20] and is associated with high morbidity and mortality [21]. In 2020, the reported preterm birth rate in Jordan was 11% [10]. Low birth weight (low, very low, and extremely low) is a predictive indicator of neonatal health. It concomitantly occurs with preterm births; therefore, it increases the long-term risk of complications. In 2020, the rate of low birth weight was 13% [22].

Although this is a sudden and unfortunate occurrence, it is important to identify a silver lining. Specifically, the pandemic provides a unique opportunity to evaluate its effects as a “natural experiment” [23]. Like the rest of the world. Jordan has been affected by the recent and ongoing changes brought about by the COVID-19 pandemic, including complete or partial lockdowns, stay-at-home orders, increased hand hygiene awareness, wearing masks, maintaining social distancing, changes in work stress, and inaccessibility of antenatal care, are likely to have had an effect on neonatal mortality, preterm birth, and low birth weight rates. This study take advantage of this unique situation and evaluate alterations in these rates, which could facilitate future studies on the underlying causes since low birth weight and preterm birth present different health outcomes when classified in different birth weight and gestation age strata. This study aimed to the assess the alterations in stillbirth, neonatal-perinatal mortality, preterm birth, and birth weight rate trends during COVID-19 national lock down. The present study is valuable as it reflects a large population in a middle-low-income country

## Methods

### Setting

On March 14^th^, 2020, the Jordanian government suspended schools, banned public gatherings, and closed borders and airports. During this period, the Ministry of Health advised the public to adhere to social distancing and proper hand hygiene. The government announced a lockdown on March 17^th,^ 2020, which subsequently turned into a strictly enforced curfew with stay-at-home orders. The more cautious relaxation measures, driven by data, were applied on 30^th^ April 2020, where the Jordanian government moved to ease the lockdown . On 5^th^ May 2020, the government issued a defense order, punishing those who do not abide by safety regulations and by wearing of masks in public with a fine. These unique conditions could pose environmental risks or benefits to pregnant women. Using a prospective maternal and newborn health registry study, we analyzed data collected from May 2019 to December 2020. Stillbirth, neonatal-perinatal mortality, gestational age of preterm births, and low birth weight trends were collected from five pilot hospitals in Jordan over the set period during the COVID-19 pandemic. Subsequently, we compared these trends with the data of pre-lockdown trends.

### Study population

Regarding the current pandemic, data were retrieved from the J-SANDS (Jordan Stillbirths and Neonatal Death Surveillance and Auditing System). The J-SANDS is a secure electronic surveillance system that was established in 2019 to collect and report standardized perinatal and neonatal mortality data from five main hospitals in Jordan. The system uses the WHO application of International Classification of Diseases, ICD-10, to classify deaths during the perinatal period (ICD-PM). These five hospitals include a university teaching hospital, a private hospital, and three public hospitals in three major governorates in Jordan. We included all births, as well as maternal demographic data, obtained from May 2019 to December 2020. We analysed data collected 11 months before the pandemic (May 2019 to March 2020) and 9 months during the pandemic (April 2020 to December 2020). The inclusion criteria were neonates born between 24 and 42 gestation weeks, with no further exclusion criteria being applied.

### Variables

Data on mothers’ socio-demographic characteristics including age, nationality, hospital birth site, income, multiple pregnancies, and parity were extracted from the JSANDS. Multiplicity was divided into singleton, twin, and triplet pregnancies, while parity included primiparous, low multiparity (having 2–4 births), and grand multiparity (having ≥ 5 births).

Prematurity was classified into extremely preterm infants (born at < 28 weeks), very preterm (born at 28–32 weeks), and moderate-to-late preterm (born at 32–37 weeks). Birth weight was divided into normal, low (< 2500 g), very low (< 1500 g), and extremely low birth weight (< 1000 g). Stillbirth was defined as delivery at ≥ 24 gestation weeks or with a birth weight of ≥ 500 g without signs of life, irrespective of death. Stillbirths include antepartum stillbirth (death before labor onset) and intrapartum stillbirth (known to be alive at labor onset). Neonatal death was defined as a live-born baby at ≥ 24 gestation weeks or with a birth weight of ≥ 500 g who died before 28 completed days after birth. In the JSANDS, neonatal deaths are divided into early neonatal death (death before 7 completed days after birth) and late neonatal death (death between 8 and 47 completed days after birth). Perinatal death was defined as fetal death after 24 gestation weeks and before 7 post-birth days.

### Ethics approval and consent to participate

This study was approved by the Institutional Review Board at Jordan University of Science and Technology (approval number 130/137/2020). To ensure data privacy, the data were exported without identifying information (e.g. name or phone number). Informed consent was obtained from all participated mothers. All methods were carried out in accordance with relevant guidelines and regulations of the Institutional Review Board of Jordan University of Science and Technology.

### Statistical analysis

Statistical analyses were performed using SPSS version 24 (IBM Corp. Armonk, NY, USA). Data were described using means (SD) and percentages. Chi-square test was used to compare percentages. Separate binary logistic regression analyses (models) were used to compare the dichotomous outcome variables (small for gestational age, stillbirth, neonatal death, and perinatal death) between the two studied periods (during the COVID-19 lockdown vs. before COVID-19). For nominal outcome variables (gestational age and birth weight), we conducted separate multinomial logistic regression analysis for each outcome to compare the outcome between the two studied periods. The main independent variable in each model was the period (during the COVID-19 lockdown vs. before COVID-19). The differences in the pregnancy outcomes between two study periods were adjusted for mother’s age, income, education, occupation, nationality, health sector, and multiplicity. These variables were selected and included in the models based on the Wald Chi-Square test statistic. A p-value of less than 0.05 was considered statistically significant.

## Results

From May 2019 to December 2020, 29592 women were admitted to the five pilot hospitals for delivery, with 31106 babies being born (15311 [49.2%] and 15795 [50.8%] before and during the COVID-19 lockdown, respectively). During the study period, 14989 (50.7%) vaginal births occurred, out of which 7228 (48.2%) and 7761 (51.8%) births occurred before and during the lockdown, respectively. Additionally, 14603 (49.3%) births occurred, by C-section out of which 7088 [48.5%] and 7515 [51.5%] were births before and during the lockdown, respectively. Table 1 presents the sociodemographic characteristics of women who delivered before and during the COVID-19 lockdown. The proportion of women with income > 500 JD was significantly lower during than that before the COVID-19 lockdown. There was no significant between-period difference in the proportion of preterm babies. Contrastingly, there was a significant between-period difference in the birth weight distribution (*p* <0.001).

**Table 1:** The characteristics of mothers who delivered before and during COVID-19 (*N*= 29592)

	Before COVID-19 (***n*** = 14316 (48.4%))		During COVID-19 (***n*** = 15276 (51.6%))		Total (***N*** = 29592)	***p***-value
	n	%	n	%	N	
Mother’s age						0.338
< 20	693	4.84	712	4.66	1405	
20–35	11162	77.97	12018	78.67	23180	
> 35	2461	17.19	2546	16.67	5007	
Nationality						0.053
Jordanian	12699	88.70	13440	87.98	26139	
Syrians	1617	11.30	1836	12.02	3453	
Sector						0.000
Public hospital	9012	62.95	10740	70.31	19752	
Private hospital	3008	21.01	2900	18.98	5908	
Teaching hospital	2296	16.04	1636	10.71	3932	
Income						0.000
≤ 700 USD	10513	79.22	12475	85.25	22988	
> 700 USD	2758	20.78	2158	14.75	4916	
Multiplicity						0.000
Singleton	13836	96.65	14379	94.13	28215	
Twin	439	3.07	835	5.47	1274	
Triplet or more	41	0.29	62	0.41	103	
Parity						0.000
Primiparous *	827	5.78	107	0.70	934	
Low multiparity (parity 2-4)	9045	63.18	10361	67.83	19406	
Grand multiparity +5	4444	31.04	4808	31.47	9252	
Gestational age						0.186
Extremely preterm (< 28 weeks)	76	0.53	77	0.50	153	
Very preterm (28 to 32 weeks)	146	1.02	161	1.05	307	
Moderate-to-late preterm (32 to 37 weeks)	1142	7.98	1117	7.31	2259	
Mode of delivery						0.595
Normal delivery	7228	48.2	7761	51.8	14989	
Caesarean section	7088	48.5	7515	51.5	14603	

Before COVID-19: May 2019 to March 2020

During COVID-19: April 2020 to December 2020

The proportion of babies born with low or very low birth weight was significantly lower during COVID-19 pandemic than that before the pandemic (Table 2). The percentage of babies with an appropriate weight for gestational age was significantly higher during, than before, the COVID-19 lockdown. There were no significant differences between the two periods in the rates of stillbirth, neonatal mortality, and perinatal mortality. The multinomial regression analysis for nominal pregnancy outcomes between two study periods were adjusted for the mother’s age, income, education, occupation, nationality, health sector, and multiplicity revealed no significant difference in the odds of extremely preterm, very preterm, and moderate-to-late preterm between the two periods (Table 3). However, babies born during the COVID-19 lockdown were significantly less likely (OR = 0.39) to be born with extremely low birth weight. In addition, there were no significant between-period differences in the rates of stillbirth, neonatal mortality, and perinatal mortality.

**Table 2:** Birth outcomes for women delivered before and during COVID-19 (*N*= 31106)

	Before COVID-19 (***n*** = 15311 (49.2%))		During COVID-19 (***n*** = 15795 (50.8%))		Total ***N*** = 31106	***p***-value
Outcome variables	n	%	n	%	N	
Birth weight						0.000
Normal birth weight	13400	87.52	13958	88.37	27358	
Low birth weight	1478	9.65	1580	10.00	3058	
Very low birth weight	200	1.31	168	1.06	368	
Extremely low birth weight	233	1.52	89	0.56	322	
Weight for GA						0.000
Appropriate for gestational age	9257	60.46	10138	64.18	19395	
Small for gestational age	1493	9.75	1686	10.67	3179	
Large for gestational age	4561	29.79	3971	25.14	8532	
Stillbirth	159	1.04	167	1.06	326	0.871
Neonatal death	221	1.46	215	1.38	436	0.539
Perinatal death	330	2.16	335	2.12	665	0.834

Before COVID-19: May 2019 to March 2020

During COVID-19: April 2020 to December 2020

**Table 3:** Logistic regression analysis of differences in birth outcomes between babies born before and during the COVID-19 lockdown (During vs. Before)

Dependent variable	OR^a^	95% confidence interval		*p*-value
Gestational age (reference: full term)				
Extremely preterm (< 28 weeks)	0.88	0.66	1.18	0.397
Very preterm (28 to 32 weeks)	0.95	0.78	1.17	0.633
Moderate-to-late preterm (32 to 37 weeks)	0.98	0.90	1.07	0.673
Birth weight (reference: normal birth weight)				
Low birth weight	1.08	0.99	1.16	0.073
Very low birth weight	0.84	0.68	1.04	0.111
Extremely low birth weight	0.39	0.30	0.50	0.000
Small for gestational age	1.07	0.98	1.17	0.121
Stillbirth (yes vs. no)	1.07	0.83	1.38	0.591
Neonatal death (yes vs. no)	0.87	0.70	1.09	0.227
Perinatal death (yes vs. no)	0.96	0.80	1.14	0.624

^a^ Adjusted for independent variables: mother’s age, income, education, occupation, nationality, health sector, and multiplicity. Gestational age and birth weight were analyzed using separate multinomial logistic regression; one model for each outcome. The outcomes (small for gestational age, stillbirth, neonatal death, and perinatal death) were analyzed using separate binary logistic regression; one model for each outcome

## Discussion

Very low birth weight (VLBW) and extremely low birth weight (ELBW) infants contribute significantly to under-5 mortality. In this context, it is important to have a better understanding of the impact of the COVID-19 pandemic on outcomes associated with preterm gestation age and low birth weight based on more appropriately standardized perinatal and neonatal mortality data. This would allow provision of more qualified care to pregnant women and the newborn. The current study reports that babies born during the period of COVID-19 were significantly less likely to be born with extremely low birth weight (0.56% vs 1.52%), even after adjusting for sociodemographic confounders. Similarly, Philip et al. reported that a significant reduction in the rate of ELBW and VLBW in Ireland includes multiple gestations (73% reduction in live births of VLBW infants and a 100% reduction of ELBW infants [24]. Their findings were reported to be possibly attributed to reduced working hours, infection avoidance due to mobility and crowding restriction, and nutritional support. In contrast, a study by Arnaez et al. from Spain [25] showed an increase in the rate of ELBW among all live births including multiple gestations during the complete lockdown period (OR: 2.21; 95% CI: 1.16–4.21; *p* = 0.016). Unexpectedly, these findings were not observed when both the lockdown and the de-escalation periods were considered. The differences in outcomes according to the period may be attributable to changes that occurred as a result of the COVID-19 pandemic, including infection prevention and control measures, the presence of around-the-clock access to emergency departments, life style and eating pattern changes, and restricted mobility of pregnant women with persistent stay home orders

At population-level reports offer conflicting data on a decrease or stability in the overall rate of preterm births during the pandemic. This study found no significant difference in the rate of preterm births, including preterm births of different gestational age strata, before and during the lockdown due to the COVID-19 pandemic. These findings are consistent with the findings of a study by Li et al. [26], conducted in Wuhan, China (9% vs 8%). In contrast with our findings, studies conducted in the United States [27] Australia [19], and the Netherlands [28], found a substantial reduction in the number of preterm births following implementation of the first national COVID-19 mitigation measures. These reductions were consistent across various degrees of prematurity. According to Been et al. [28], the reduction in the prematurity rates was probably due to a combination of factors including stopping work, increased hygiene measures, social distancing resulting in fewer infections by common pathogens, and less air pollution. Similarly, additional national studies done in Ireland and Denmark showed a significant decrease in the rate of extremely preterm births since the start of the COVID-19 pandemic [24, 29]. No change was reported in the incidence of preterm births in four hospitals in the United States [30], and one hospital in London [31].

Riley et al. [32] reported that a reduction of as little as 10% in the provision of antenatal health services in low- and middle-income countries during the COVID-19 pandemic, could lead to an increase in the rates of maternal and neonatal mortality, an increase in the number of women suffering from major obstetric complications, and an increase in newborns with major health-problems being deprived of suitable care. In Jordan, Muhaidat et al. [33] reported a significant increase in the percentage of pregnant women who did not receive antenatal care during the pandemic (from 4% to 59.5%), due to national mitigation measures in Jordan.

In this study, we did not observe differences between the two periods in neonatal mortality, perinatal mortality, or stillbirth. Inconsistent with this finding, a Nepalese study showed an increase in stillbirth and neonatal mortality during the COVID-19 pandemic compared with before [34]. Furthermore, middle-to-low-income countries reported a slight decrease in neonatal mortality, which could be attributed to increased severity of maternal infection or preterm birth [35], whereas we found no significant increase in prematurity.

To our knowledge, our study is by far the largest to have assessed the impact of COVID-19 mitigation measures on birth outcomes in a middle-income country. Given the large sample size of our study (*n*=31,106), our findings are representative of Jordan’s population and could facilitate further studies on the effect of cumulative socioenvironmental and maternal behavioral alteration on the factors contributing to extremely low birth weight, which has adverse effects on neonatal wellbeing and added long-term morbidity during the coronavirus pandemic [36]. Additionally, our findings could facilitate comparison with other data worldwide to elucidate the significance of these findings and the underlying causes of extremely low birth weight to minimize undesired future outcomes.

Our study has several limitations. First, we could not compare our results to previous Jordanian findings since the J-SANDS was only recently established in August 2019. A longer analysis period could have yielded more reliable rates since Jordan has a limited data system in place documenting stillbirths and other neonatal outcomes.

Second, the J-SANDS only includes five hospitals in Jordan, which could limit the generalizability of the results nationwide.

## Conclusion

After adjustment for sociodemographic factors, the study found that there were significant differences in the populations of the two periods in terms of the rate of extreme low birth weight infants, but no difference in the rates of preterm birth, neonatal-perinatal death, and stillbirth. To see if the changes persist, birth outcomes must be tracked throughout the second year of the COVID-19 pandemic and beyond. Future epidemiological and experimental research is needed to better understand the effects of socioeconomic, maternal behavioral, and nutritional factors on the variable reported birth outcomes that occurred worldwide during the pandemic in order to develop effective preventive strategies.

## Data Availability

The datasets generated and/or analysed during the current study are not publicly available due to limitations of ethical approval involving the patient data and anonymity but are available from from yskhader@just.edu.jo on reasonable request.

## Abbreviations

COVID-19: Coronavirus disease 2019
ELBW: Extremely low birth weight
ICD-10: International Classification of Diseases-10
ICD-PM: WHO application of ICD-10 to perinatal mortality
J-SANDS: Jordan stillbirths and neonatal death surveillance and auditing system
SARS-CoV-2: Severe acute respiratory syndrome coronavirus 2
